# Full-Genome Sequences and Phylogenetic Analysis of Archived Danish European Bat Lyssavirus 1 (EBLV-1) Emphasize a Higher Genetic Resolution and Spatial Segregation for Sublineage 1a

**DOI:** 10.3390/v13040634

**Published:** 2021-04-07

**Authors:** Sten Calvelage, Conrad M. Freuling, Anthony R. Fooks, Dirk Höper, Denise A. Marston, Lorraine McElhinney, Thomas Bruun Rasmussen, Stefan Finke, Martin Beer, Thomas Müller

**Affiliations:** 1 Institute of Diagnostic Virology, Friedrich-Loeffler-Institut (FLI), 17493 Greifswald-Insel Riems, Germany; sten.calvelage@fli.de (S.C.); dirk.hoeper@fli.de (D.H.); martin.beer@fli.de (M.B.); 2 Central Duties, Friedrich-Loeffler-Institut (FLI), 17493 Greifswald-Insel Riems, Germany; conrad.freuling@fli.de; 3 Animal & Plant Health Agency, WHO Collaborating Centre for Rabies Surveillance and Research, OIE Reference Laboratory for Rabies, Weybridge KT15 3NB, Surrey, UK; Tony.Fooks@apha.gov.uk (A.R.F.); denise.marston@apha.gov.uk (D.A.M.); Lorraine.McElhinney@apha.gov.uk (L.M.); 4 Virus & Mikrobiologisk Specialdiagnostik, Statens Serum Institut, 2300 Copenhagen, Denmark; tbru@ssi.dk; 5 Institute of Molecular Virology and Cell Biology, Friedrich-Loeffler-Institut (FLI), WHO Collaborating Centre for Rabies Surveillance and Research, OIE Reference Laboratory for Rabies, 17493 Greifswald-Insel Riems, Germany; stefan.finke@fli.de

**Keywords:** European bat lyssavirus 1 (EBLV-1), next generation sequencing (NGS), bats, full-genome sequencing, zoonoses

## Abstract

European bat lyssavirus type 1 (EBLV-1) is the causative agent for almost all reported rabies cases found in European bats. In recent years, increasing numbers of available EBLV-1 full genomes and their phylogenetic analyses helped to further elucidate the distribution and genetic characteristics of EBLV-1 and its two subtypes, namely EBLV-1a and EBLV-1b. Nonetheless, the absence of full-genome sequences from regions with known detections of EBLV-1 still limit the understanding of the phylogeographic relations between viruses from different European regions. In this study, a set of 21 archived Danish EBLV-1 samples from the years 1985 to 2009 was processed for the acquisition of full-genome sequences using a high-throughput sequencing approach. Subsequent phylogenetic analysis encompassing all available EBLV-1 full genomes from databases revealed the Danish sequences belong to the EBLV-1a subtype and further highlighted the distinct, close phylogenetic relationship of Danish, Dutch and German isolates in this region. In addition, the formation of five putative groups nearly exclusively formed by Danish isolates and the overall increased resolution of the EBLV-1a branch indicate a higher genetic diversity and spatial segregation for this sublineage than was previously known. These results emphasize the importance of phylogenetic analyses of full-genome sequences of lyssaviruses for genetic geography.

## 1. Introduction

Bats (Chiroptera) are suggested to represent reservoirs for almost all current members of the lyssavirus genus [1,2], which constitutes of 17 recognized member species and 1 related, yet unclassified virus [3,4]. In contrast to classical rabies virus (RABV) that has a global distribution, non-RABV lyssaviruses have a more limited geographic and host range distribution [5]. Although lyssavirus infections of bats have been documented in many parts of the world, different virus species are present in different regions and infect particular species of bats that serve as reservoirs only [6]. Whereas RABV is only associated with bats in the Americas, the remaining lyssaviruses predominate in a species dependent manner across all other continents except Antarctica [7].

*The European bat 1 lyssavirus* is one of six bat-associated lyssavirus member species so far reported in Europe [8]. As the most prevalent lyssavirus found in European bats EBLV-1 is primarily associated with the Serotine bat (*Eptesicus serotinus*) and its closely related species the Meridional serotine (*Eptesicus isabellinus*) as its main host species [9,10] with confirmed spillover infections into other mammalian species including stone marten (*Martes fonia*) [11], sheep (*Ovis aries*) [12] and cat (*Felis catus*) [13]. Even though only a small number of incidents has been reported so far, the potential threat to human life due to a bat-transmitted EBLV-1 infection is still present, as a confirmed fatal case in the Ukraine [14] demonstrated the susceptibility of humans for the virus. Several studies investigated the genetic characteristics and phylogenetic relationship of EBLV-1 isolates in a number of countries, exposing some key features of the virus and its distribution in Europe [15,16,17,18,19,20,21]. As a result, phylogenetic analyses of partial [16,21,22] and full-genome sequences [15] revealed a separation of the EBLV-1 isolates into two distinct subtypes, namely EBLV-1a and 1b, with an expansion along an east–west axis for EBLV-1a and a north–south axis for EBLV-1b and partial overlapping of those two areas of distribution [16].

Denmark ranks among the three European countries with the highest case numbers of bat associated rabies cases; only Germany and the Netherlands have higher case figures [23,24]. Since the first detection of bat rabies in Denmark in the mid-1980s [23], a total of 226 cases of bat rabies have been reported to the Rabies Bulletin Europe [25] (https://www.who-rabies-bulletin.org, assessed 6 April 2021), of which the great majority is assumed to have been caused by EBLV-1. However, despite this plethora of Danish bat rabies cases only few partial and full genome sequences were previously available [15,16,21,22]. As a result, the genetic diversity of EBLV-1 isolates from the northernmost known distribution area of its reservoir host has only been partly reflected in phylogenetic analyses.

In this study we obtained full-genome sequences from a panel of archived Danish EBLV-1 samples collected over a period of 24 years, to gain further insights into the genetics of EBLV-1. The subsequent phylogenetic analysis and spatio-genetic mapping of the newly generated Danish sequences contribute to a better understanding of the EBLV-1 genetic diversity, its geographic pattern and spatio-temporal correlations between geographically close isolates.

## 2. Materials and Methods

### 2.1. Sampling

A total of 21 archived Danish EBLV-1 positive brain samples originating from 19 Serotine bats (*Eptesicus serotinus*), one Soprano pipistrelle (*Pipistrellus pygmaeus*) and one spillover case in sheep (*Ovis aries*) were included for genetic and phylogenetic analysis. The samples were collected in 4 of the 5 regions of Denmark including Syddanmark, Midtjylland, Nordjylland and Sjælland (Table 1) between 1985 and 2009. Information on geographic location and year was collected if available.

### 2.2. Sample Preparation

The brain samples were processed according to the workflow for the NGS-based metagenomic pathogen detection published by Wylezich et al. [26]. In short, an estimated amount of 20 mg original brain material was combined with 1 mL Trizol and subsequently disintegrated using the TissueLyser followed by a thermal inactivation step. For samples from cell culture supernatant, mixtures of 250 µL sample volume and 750 µL Trizol were prepared for RNA extraction which was realized by the RNeasy Mini Kit (Qiagen, Hilden, Germany) and on-column DNAse I digestion. For cDNA synthesis, 500 ng of sample RNA was used in.

The cDNA synthesis system kit (Roche Diagnostics, Rotkreuz, Switzerland) together with random hexamer primers. The resulting double-stranded cDNA was further processed by ultrasonic fragmentation (Covaris M220-500 bp mode) followed by library preparation utilizing the Gene Read L Core Kit (Qiagen) and IonXpress Barcode Adaptors (Thermo Fisher Scientific, Waltham, MA, USA). After quality control and quantification of the generated libraries, samples were sequenced on an Ion S530 chip in 400 bp mode with an IonTorrent S5 XL system according to the manufacturer’s instructions.

For sample 34702DEN (Table 1), sample disintegration, RNA extraction and cDNA synthesis were conducted as described above. For the generation of the Illumina compatible library, the SPRI-TE instrument with SPRIworks II cartridges (Beckman Coulter, Fullerton, CA, USA) was utilized in combination with appropriate adapters. After quantification and quality control, sequencing was realized on an Illumina MiSeq platform using MiSeq reagent kit v3 (Illumina, San Diego, CA, USA) in 2 × 300 bp paired end mode.

### 2.3. Genome Assembly and Phylogenetic Analysis

For the generation of full-genome sequences from the examined EBLV-1 samples, sequencing data were automatically adapter trimmed by the Ion Torrent Software Suite (v.5.12.1) and the resulting raw reads were used for mapping against the EBLV-1 reference sequence (NC_009527) [18] utilizing the 454 Sequencing System Software v3.0 (Roche). Subsequently, full or partial mapped reads were considered for de novo assembly (454 Sequencing System Software v3.0, Roche) and the generated full-genome sequences were annotated via Geneious Prime (2019.2.3; build 24 September 2019). All obtained full-genome sequences were submitted to the European Nucleotide Archive (ENA) under the study accession PRJEB42002. In preparation of the phylogenetic analysis, a total of 90 available EBLV-1 full-genome sequences were selected based on the datasets used in two previously published studies [15,17] (Table S1). Together with the 21 newly generated Danish EBLV-1 as well as a Duvenhage lyssavirus (DUVV) reference sequence (NC_020810) [27] as defined outgroup, the investigated dataset encompassed a total of 112 sequences. The complete dataset was aligned using the MAFFT algorithm (v.7.450) [28] with default settings as implemented in Geneious Prime. Phylogenetic analyses were realized using the IQ-Tree-Software (v.1.6.5) [29] and 100.000 ultrafast-bootstrap [30] for maximum-likelihood phylogenetic tree construction under usage of the ModelFinder [31] feature for best-fit nucleotide substitution model selection (GTR + F + I + Γ_4_).

## 3. Results

### 3.1. Comparison of Full-Length Sequences

For the 21 archived Danish EBLV-1 samples, partial and full genomes could be obtained with sequence lengths ranging between 11925 and 11967 base pairs (Table 1). Missing sequence information was limited to the 3′ and 5′ untranslated regions while intra- and intergenomic regions could be completely assembled for all samples. An alignment of the obtained 21 Danish genomes revealed a nucleotide sequence identity of 99.5 %. This high genetic homogeneity of the examined Danish virus genomes over a 24-year period is in accordance with previous findings of low substitution rates described for EBLV-1 [15,16]. Furthermore, the alignment highlighted the high amount of synonymous nucleotide exchanges found between single Danish cases and the consensus of all Danish EBLV-1 sequences. In contrast, only a low number of nonsynonymous exchanges could be observed (Figure S1).

### 3.2. Phylogenetic Analysis Including New Danish EBLV-1 Sequences Reveal Higher Genetic Resolution for the A1 Cluster of Sublineage 1a

**Figure 1:**
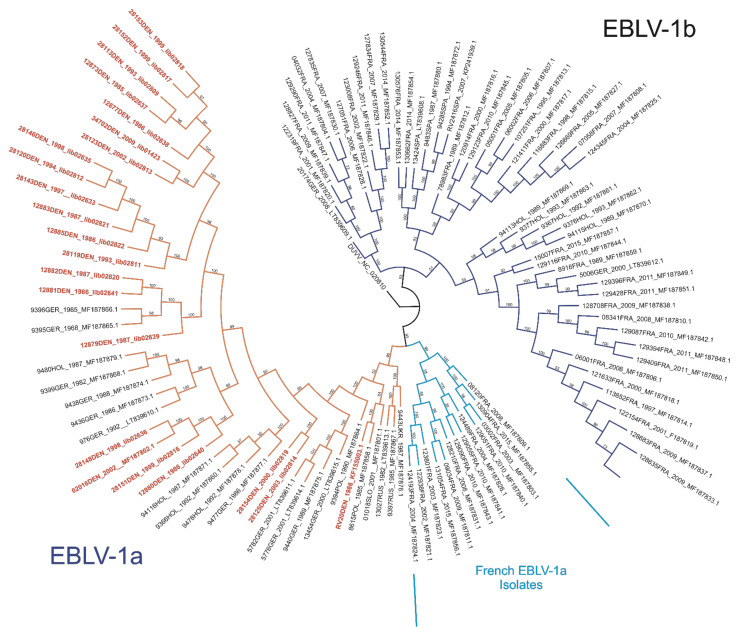
Circular phylogenetic tree of 112 EBLV-1 full-genome sequences originating from 9 European countries including Spain, France, Poland, Slovakia, Russia, Ukraine, Germany, The Netherlands and Denmark using isolate DUVV_Ref_Seq_NC_020810 as defined outgroup. Ultrafast bootstrap values (percentage) are indicated at each branch. Sublineage EBLV-1a (orange branches) and 1b (dark blue branches) are clearly separated. The distinct cluster formed by French isolates within the EBLV-1a sublineage is indicated in turquoise. EBLV-1 full genome sequences from Denmark are highlighted in red.

**Figure 2:**
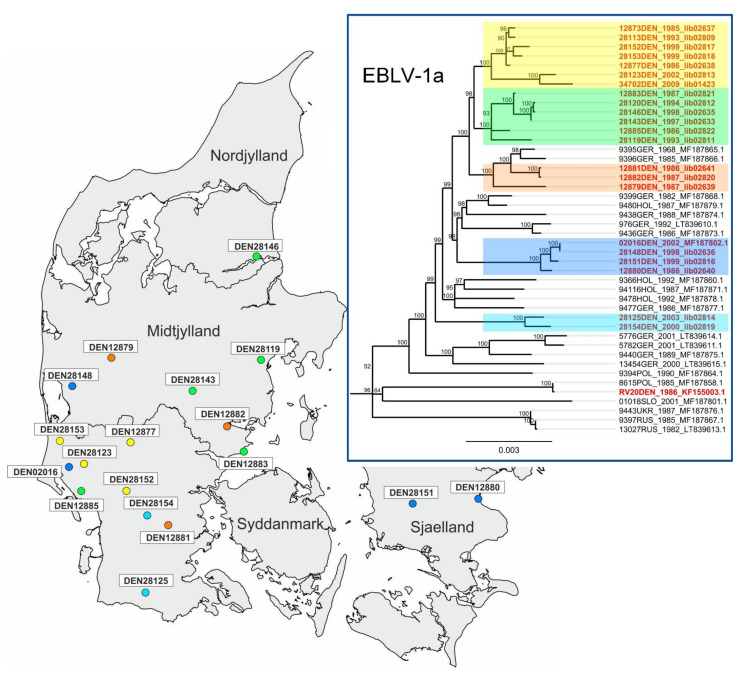
Map of Denmark showing the geographic location of 17 of the 21 characterized Danish EBLV-1 isolates and one of the two previously described Danish isolates available in Genbank (02016DEN). For five Danish EBLV-1 isolates no information on the geographic location was available. Insert upper right corner: Reduced maximum likelihood phylogenetic tree of sublineage EBLV-1a (N = 45) with the French EBLV-1a isolates forming a distinct cluster (Figure 1) excluded for better visualization. Ultrafast bootstrap values (percentage) are indicated at each node and branches are scaled based on the number of substitutions per site. The affiliation of the EBLV-1 isolates to one of the five putative A1 subclades of Danish origin are highlighted in different colors with yellow, green, orange, blue and cyan representing isolates from Groups 1, 2, 3, 4, and 5, respectively (Table 2).

The phylogenetic analysis comprising the 21 Danish and an additional 90 EBLV-1 full-genome sequences from nine different European countries resulted in a strict genetic segregation of the EBLV-1 isolates into two sublineages, namely EBLV-1a and 1b (Figure 1) as described previously [15,16,17,21]. The archived Danish isolates grouped exclusively in the A1 cluster of sublineage EBLV-1a, confirming previous analyses of the only two Danish EBLV-1 full genome sequences available in genome databases (Figure 1 and Figure 2, RV20DEN and 02016DEN) [15,17]. Together with isolates originating from the Netherlands, Germany, Poland, Slovakia, Ukraine and Russia the sequences broadly diversified in distinct A1 subclades, while the French EBLV-1a isolates all showed a characteristic drift by forming an exclusive distinct phylogenetic cluster (A2) within the same sublineage (Figure 1, turquoise clade). The Danish EBLV-1a isolates did not cluster in a distinct monophyletic group (Figure 1) but segregated in five of eleven manifesting phylogenetic subclades in cluster A1 as supported by high bootstraps values (Figure 2, Table 2). On closer analysis, all but one isolate showed close genetic relationship with German and Dutch isolates including the previously published 2016DEN sequence [15,17]. In contrast, isolate RV20DEN [32] (Figure 1 and Figure 2) seems to share a higher sequence identity with Polish and Slovakian isolates. However, the weak branch support for the classification of the RV20DEN isolate as indicated by low bootstrap values, makes a final assessment of the phylogenetic relationship difficult (Figure 2).

### 3.3. Genetic Geography Reveals Small-Scale Spatial Distribution of Danish EBLV-1a Isolates

A spatio-genetic assessment of EBLV-1a sequences taking the geographic location of the Danish isolates into account revealed a time independent small-scale spatial distribution (Figure 2, Table 2).

Groups 1 (Figure 2; yellow) and 5 (cyan) showed the strongest correlation between their phylogenetic relation and geographic location. Representatives from Group 1 detected between 1986 and 2009 originated from a rather small area in the northwestern part of the Syddanmark region within a maximum distance of 50 km^2^ between isolates, while those of Group 5 exhibited a similar pattern. Group 5 is the most basal phylogenetic group of all Danish EBLV-1 consisting of two isolates from the years 2000 (28154DEN) and 2003 (28125DEN), both identified in southern Jutland (Syddanmark region) close to the border of Germany (Figure 2). In contrast, EBLV-1a isolates from Groups 2–4 (Figure 2; green, orange, blue) seemed to have a wider geographical but overlapping distribution within Denmark with isolates found in more than one Danish region. Danish Group 2 and 3 EBLV-1a isolates obtained between 1986 and 1998 were found in 3 of the 5 Danish regions (Syddanmark, Midtjylland and Nordjylland) on the Jutland peninsula with a roughly equivalent geographic coverage. In comparison to the monophyletic groups formed by all other newly generated Danish EBLV-1 sequences, group 3 represented a paraphyletic group that is highly related to two previously investigated EBLV-1 isolates from the northern region of Germany (9395GER—Hamburg 1968 and 9396GER—Rostock 1985 [9]). In contrast, Group 4 EBLV-1a isolates showed the highest geographical distances between locations among all groups with two cases found in the western part of Jutland (Figure 2, 02016DEN—2002; 28148DEN—1998, blue data points) and two cases positioned in the central and eastern areas of the Sjælland region (28151DEN—1999; 12880DEN—1986), resulting in a maximum linear distance of 247 km between isolates 28148DEN and 12880DEN.

## 4. Discussion

With 19 out of 21 samples, the vast majority of the investigated Danish cases is constituted by samples of *E. serotinus* (Table 1), the main reservoir of EBLV-1 [9,10] giving further evidence for the high affiliation of EBLV-1 to this particular bat species. Interestingly, EBLV-1 has also been sporadically detected in other bat species including *Pipistrellus, Myotis, Rhinopholus, Mineopterus, Nyctalus and Barbastella* spp. [10,33]. Here, we report an EBLV-1 case in a Soprano pipistrelle bat (*Pipistrellus pygmaeus*). Generally, those cases are considered spillover events rather than independent infections cycles. Early publications from Denmark also indicated rabies cases in Daubenton’s bats (*Myotis daubentonii*) and Pond bats (*Myotis dasycneme*) [23] giving reason to believe that next to EBLV-1 also other lyssaviruses including EBLV-2 are circulating in Danish bats. Considering the high number of reported bat rabies cases (N = 226) from Denmark on a European scale over the past 35 years [23], it is unfortunate that only 21 EBLV-1 isolates collected over a time span of 24 years were available for genetic characterization.

Using a larger panel of EBLV-1 full genome sequences from the Danish part of the northernmost range of its main reservoir host we corroborate previous findings on the segregation of this virus species into two lineages, e.g., EBLV-1a and -1b [16,19,21,22,34,35], with the Danish isolates grouping exclusively within sublineage 1a (Figure 1 and Figure 2). Interestingly, for reasons unknown, this genetic segregation and dispersal in Europe is not reflected by the genetic diversity observed in the reservoir host, the Serotine bat [15,36].

The cause of the relatively high genetic homogeneity among EBLV-1a isolates across Europe compared to EBLV-1b is still unknown [16]. While analyses of deep phylogenetic relationships among virus isolates are limited by the number of full-genome sequences available, with the inclusion of a larger panel of Danish isolates we could demonstrate that sublineage 1a is more divergent than previously thought, though at a finer phylogenetic resolution as opposed to sublineage 1b (Figure 1) [16] Based on high bootstrap values, our analysis further supports the subdivision of cluster A1 of sublineage EBLV-1a into at least eleven putative subclades (Figure 1) of which five subclades mainly comprise of sequences from Danish isolates (Figure 2).

Previous analyses demonstrated a less pronounced geographic clustering of EBLV-1a isolates in comparison to EBLV-1b [15,16]. However, here further evidence is provided that the higher genetic resolution also allows for a better geographic assignment of isolates within the cluster level (A1, A2) of sublineage 1a. Even though several EBLV-1a subclades are likely to occur seemingly small-clustered in one and the same region, their geographical distribution may be more or less overlapping (Figure 2) as hypothesized previously [19]. This pattern might be a result of the migration behavior of *E. serotinus*, which is considered to be a sedentary bat species only occasionally performing dispersal flights. The distances between summer and winter roost are often shorter than 40–50 km with recorded maximum movements between 107 and 142 km [36,37].

The close relationship of Danish with German and Dutch isolates both genetically and geographically is in accordance with previous phylogenetic analysis based on the N and G gene sequences [16] and strongly suggests a regionally restricted virus transmission between populations of *E. serotinus* in this particular region [15,16]. The fact that isolate RV20DEN is rooted at a more basal position in the phylogenetic tree together with Polish and Slovakian EBLV-1a isolates seems to contradict this assumption, however, the grouping of the isolate is impeded by a low branch support.

This study confirms previous observations that the small-scale spatial distribution of EBLV-1a variants is time independent [19], as EBLV-1 isolates were scattered mainly over the Jutland peninsula without revealing distinct geographic hotspots or temporal dependencies. However, the genetic distance of EBLV-1a isolates rather increases linearly with increasing distance in time [19]. A reasonable explanation for that might be the overall low substitution rate known for EBLV-1 [15,16] and the resulting high sequence identity of EBLV-1 genomes, which is maintained over long time periods as it was also shown for the Danish isolates in this study. A similar effect was recently described in a comprehensive study encompassing available and newly generated full-genome sequences for the European bat lyssavirus type 2 (EBLV-2) [38]. Therefore, even higher numbers of EBLV-1 full-genome sequences might not lead to a better genetic resolution on the country level but will likely further improve the genetic subdivision within the EBLV-1a sublineage at a supra-regional level. In this respect, the generation of further full-genome sequences in particular from European regions not covered yet should be envisaged to further elucidate the more basal structure of the phylogenetic tree for the EBLV-1a cluster, possibly also resolving the conundrum of the RV20DEN isolate.

## Figures and Tables

**Table 1:** Overview of the investigated Danish samples.

Sample ID	Library No.	Species	Sample Matrix	Year of Collection	Location	Sequence Length
12873DEN	2637	Bat (*E. serotinus*)	Brain	1985	n.d.	11,963
12877DEN	2638	Bat (*E. serotinus*)	Brain	1986	Grindsted	11,925
12879DEN	2639	Bat (*E. serotinus*)	Brain	1987	Vildbjerg	11,966
12880DEN	2640	Bat (*E. serotinus*)	Brain	1986	Køge	11,945
12881DEN	2641	Bat (*E. serotinus*)	Brain	1986	Sommersted	11,953
12882DEN	2820	Bat (*E. serotinus*)	Brain	1987	Horsens	11,953
12883DEN	2821	Bat (*E. serotinus*)	Brain	1987	Juelsminde	11,934
12885DEN	2822	Bat (*E. serotinus*)	Brain	1986	Esbjerg	11,955
28113DEN	2809	Bat (*E. serotinus*)	Brain	1993	n.d.	11,967
28119DEN	2811	Bat (*E. serotinus*)	CCS	1993	Arhus	11,965
28120DEN	2812	Bat (*E. serotinus*)	CCS	1994	Arhus	11,958
28123DEN	2813	Bat (*E. serotinus*)	CCS	2002	Varde	11,966
28125DEN	2814	Bat (*E. serotinus*)	CCS	2003	Bylderup	11,965
28143DEN	2633	Bat (*E. serotinus*)	CCS	1997	Bryrup	11,966
28146DEN	2635	Bat (*P. Pygmaeus*)	CCS	1998	Hadsund	11,966
28148DEN	2636	Sheep (*Ovis aries*)	CCS	1998	Lem	11,966
28151DEN	2816	Bat (*E. serotinus*)	CCS	1999	Zealand Soro	11,965
28152DEN	2817	Bat (*E. serotinus*)	CCS	1999	Holsted	11,966
28153DEN	2818	Bat (*E. serotinus*)	CCS	1999	Nebel	11,966
28154DEN	2819	Bat (*E. serotinus*)	CCS	2000	Rodding	11,964
34702DEN	1423	Bat (*E. serotinus*)	CCS	2009	n.d.	11,967 *

CCS—Cell Culture Supernatant; * sequence for 34720DEN in separate samples processing step acquired.

**Table 2:** Groups determined for the 21 Danish EBLV-1 full genomes and the isolate 02016DEN based on their phylogenetic relationship.

Group (Color)	Sample ID	Host Species	Region	Year
1 (yellow)	12873DEN	Serotine Bat	-	1985
	28113DEN	Serotine Bat	-	1993
	28152DEN	Serotine Bat	Syddanmark	1999
	28153DEN	Serotine Bat	Syddanmark	1999
	12877DEN	Serotine Bat	Syddanmark	1986
	28123DEN	Serotine Bat	Syddanmark	2002
	34702DEN	Serotine Bat	-	2009
2 (green)	12883DEN	Serotine Bat	Midtjylland	1987
	12885DEN	Serotine Bat	Syddanmark	1986
	28119DEN	Serotine Bat	Midtjylland	1993
	28120DEN	Serotine Bat	Midtjylland	1994
	28143DEN	Serotine Bat	Midtjylland	1997
	28146DEN	Soprano Pipistrelle	Nordjylland	1998
3 (orange)	12879DEN	Serotine Bat	Midtjylland	1987
	12881DEN	Serotine Bat	Syddanmark	1986
	12882DEN	Serotine Bat	Midtjylland	1987
4 (blue)	02016DEN *	Serotine Bat	Syddanmark	2002
	12880DEN	Serotine Bat	Sjælland	1986
	28148DEN	Sheep	Midtjylland	1998
	28151DEN	Serotine Bat	Sjælland	1999
5 (cyan)	28125DEN	Serotine Bat	Syddanmark	2003
	28154DEN	Serotine Bat	Syddanmark	2000

* GenBank MF187802.1.

## Data Availability

All generated full-genome sequences were submitted to the European Nucleotide Archive (ENA) under the study accession PRJEB42002.

## Supplementary Materials

The following are available online at https://www.mdpi.com/article/10.3390/v13040634/s1, Table S1: Listing of additional EBLV-1 full-genome sequences considered for the phylogenetic analysis that were not already included in database accessible EBLV-1 full genomes previously covered by Troupin et al. [15]. Figure S1: Schematic illustration of the EBLV-1 genome.
