# Supplementary material for: Full-Genome Sequences and Phylogenetic Analysis of Archived Danish European Bat Lyssavirus 1 (EBLV-1) Emphasize a Higher Genetic Resolution and Spatial Segregation for Sublineage 1a
Source: Viruses. 2021 Apr 7;13(4):634. doi: 10.3390/v13040634 (PMC8067844; doi:10.3390/v13040634)
Supplement: Supplementary file 1 [file viruses-13-00634-s001.pdf]

Supplementary File

Full-genome Sequences and Phylogenetic Analysis of Archived Danish European Bat 1 Lyssaviruses Emphasize a Higher Genetic Resolution and Spatial Segregation for Sublineage 1a

Sten Calvelage, Conrad M. Freuling, Anthony R. Fooks, Dirk Höper, Denise A. Marston, Lorraine McElhinney, Thomas Bruun Rasmussen, Stefan Finke, Martin Beer and Thomas Müller

Table S1. Listing of additional EBLV-1 full- genome sequences considered for the phylogenetic analysis that were not already included in database accessible EBLV-1 full-genomes previously covered by Troupin *et al.* [1].

Sample	Sequence/ ENA Project Accession	Country	Year	Longitude	Latitude	Reference
13027RUS	LT839613.1	Russia	1982	-	-	[2]
13424SPA	LT839608.1	Spain	1989	-	-	[2]
13454GER	LT839615.1	Germany	2000	-	-	[2]
20174GER	LT839609.1	Germany	2008	-	-	[2]
5006GER	LT839612.1	Germany	2000	-	-	[2]
5776GER	LT839614.1	Germany	2001	-	-	[2]
5782GER	LT839611.1	Germany	2001	-	-	[2]
976GER	LT839610.1	Germany	1992	-	-	[2]
12873DEN	PRJEB42002	Denmark	1985	-	-	This study
12877DEN	PRJEB42002	Denmark	1986	8.944	55.7544	This study
12879DEN	PRJEB42002	Denmark	1987	8.7660	56.1954	This study
12880DEN	PRJEB42002	Denmark	1986	12.1821	55.4575	This study
12881DEN	PRJEB42002	Denmark	1986	9.2953	55.3183	This study
12882DEN	PRJEB42002	Denmark	1987	9.8434	55.838	This study
12883DEN	PRJEB42002	Denmark	1987	9.9988	55.706	This study
12885DEN	PRJEB42002	Denmark	1986	8.4852	55.4985	This study
28113DEN	PRJEB42002	Denmark	1993	-	-	This study
28119DEN	PRJEB42002	Denmark	1993	10.1585	56.1845	This study
28120DEN	PRJEB42002	Denmark	1994	10.1585	56.1845	This study
28123DEN	PRJEB42002	Denmark	2002	8.5118	55.6414	This study
28125DEN	PRJEB42002	Denmark	2003	9.0835	54.9592	This study
28143DEN	PRJEB42002	Denmark	1997	9.5223	56.0231	This study
28146DEN	PRJEB42002	Denmark	1998	10.1167	56.7167	This study
28148DEN	PRJEB42002	Denmark	1998	8.4019	56.0481	This study
28151DEN	PRJEB42002	Denmark	1999	11.5709	55.4322	This study
28152DEN	PRJEB42002	Denmark	1999	8.9062	55.499	This study
28153DEN	PRJEB42002	Denmark	1999	8.2868	55.7621	This study
28154DEN	PRJEB42002	Denmark	2000	9.0996	55.3696	This study
34702DEN	PRJEB42002	Denmark	2009	-	-	This study

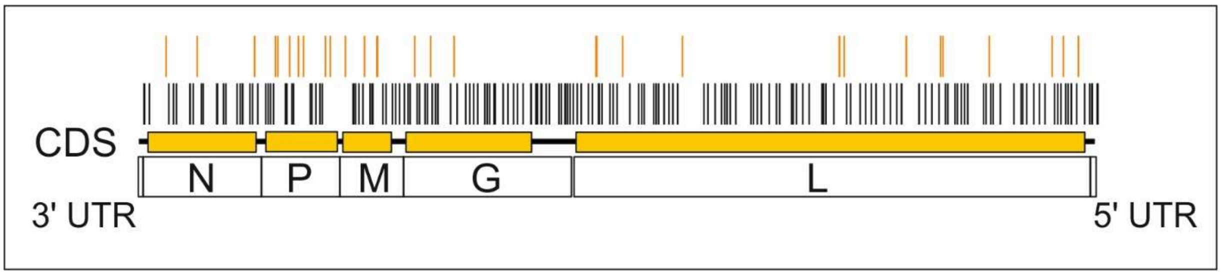

Figure S1 Schematic illustration of the EBLV-1 genome. Lines indicate the position of nucleotide exchanges observed between the Danish EBLV-1 consensus sequence (obtained by the alignment of all generated Danish EBLV-1 sequences) and single Danish cases (see Table 1). Black lines represent synonymous mutations whereas orange lines represent nonsynonymous mutations.

References

1. Troupin, C.; Picard-Meyer, E.; Dellicour, S.; Casademont, I.; Kergoat, L.; Lepelletier, A.; Dacheux, L.; Baele, G.; Monchâtre-Leroy, E.; Cliquet, F.; et al. Host Genetic Variation Does Not Determine Spatio-Temporal Patterns of European Bat 1 Lyssavirus. *Genome Biol. Evol.* **2017**, *9*, 3202–3213, doi:10.1093/gbe/evx236.
2. Eggerbauer, E.; Pfaff, F.; Finke, S.; Höper, D.; Beer, M.; Mettenleiter, T.C.; Nolden, T.; Teifke, J.-P.; Müller, T.; Freuling, C.M. Comparative analysis of European bat lyssavirus 1 pathogenicity in the mouse model. *PLoS Negl. Trop. Dis.* **2017**, *11*, e0005668, doi:10.1371/journal.pntd.0005668.
